# ADAM17 and EGFR regulate IL‐6 receptor and amphiregulin mRNA expression and release in cigarette smoke‐exposed primary bronchial epithelial cells from patients with chronic obstructive pulmonary disease (COPD)

**DOI:** 10.14814/phy2.12878

**Published:** 2016-08-25

**Authors:** Marta Stolarczyk, Gimano D. Amatngalim, Xiao Yu, Mieke Veltman, Pieter S. Hiemstra, Bob J. Scholte

**Affiliations:** ^1^ Cell BiologyErasmus MCRotterdamThe Netherlands; ^2^ PulmonologyLeiden University Medical Center (LUMC)LeidenThe Netherlands

**Keywords:** A disintegrin and metalloprotease 17 (ADAM17), amphiregulin (AREG), Chronic Obstructive Pulmonary Disease (COPD), epidermal growth factor receptor (EGFR), IL6 receptor (IL6R), TACE

## Abstract

Aberrant activity of a disintegrin and metalloprotease 17 (ADAM17), also known as TACE, and epidermal growth factor receptor (EGFR) has been suggested to contribute to chronic obstructive pulmonary disease (COPD) development and progression. The aim of this study was to investigate the role of these proteins in activation of primary bronchial epithelial cells differentiated at the air–liquid interface (ALI‐PBEC) by whole cigarette smoke (CS), comparing cells from COPD patients with non‐COPD. CS exposure of ALI‐PBEC enhanced ADAM17‐mediated shedding of the IL‐6 receptor (IL6R) and the EGFR agonist amphiregulin (AREG) toward the basolateral compartment, which was more pronounced in cells from COPD patients than in non‐COPD controls. CS transiently increased IL6R and AREG mRNA in ALI‐PBEC to a similar extent in cultures from both groups, suggesting that posttranslational events determine differential shedding between COPD and non‐COPD cultures. We show for the first time by in situ proximity ligation (PLA) that CS strongly enhances interactions of phosphorylated ADAM17 with AREG and IL‐6R in an intracellular compartment, suggesting that CS‐induced intracellular trafficking events precede shedding to the extracellular compartment. Both EGFR and ADAM17 activity contribute to CS‐induced IL‐6R and AREG protein shedding and to mRNA expression, as demonstrated using selective inhibitors (AG1478 and TMI‐2). Our data are consistent with an autocrine‐positive feedback mechanism in which CS triggers shedding of EGFR agonists evoking EGFR activation, in ADAM17‐dependent manner, and subsequently transduce paracrine signaling toward myeloid cells and connective tissue. Reducing ADAM17 and EGFR activity could therefore be a therapeutic approach for the tissue remodeling and inflammation observed in COPD.

## Introduction

Chronic obstructive pulmonary disease (COPD) is a progressive lung disorder characterized by irreversible airflow obstruction due to airway inflammation, infection, and tissue remodeling (Vestbo et al. 2013). Airway epithelial cells play a central role in the pathogenesis of COPD through a variety of mechanisms, including production of inflammatory mediators, antimicrobial peptides, and growth factors (Hiemstra et al. 2015). Exposure of the lung tissue to triggers like cytotoxic particles and gasses, including cigarette smoke, microbes, and innate immune mediators (Koff et al. 2008; Kim et al. 2011) (Lemjabbar‐Alaoui et al. 2011) (Shao 2004) (Zhou et al. 2012) activate various matrix metalloproteinases (MMPs) and a disintegrin and metalloproteinases (ADAMs) expressed by airway epithelial cells (Dijkstra et al. 2009; Paulissen et al. 2009). The activity of MMPs and ADAMs contributes not only to proteolytic degradation of lung tissue, but also to regulation and processing of numerous receptor activating proteins (Richter et al. 2002; Gomez et al. 2005; Bell et al. 2007; Baumgart et al. 2010). Through these various activities, MMPs and ADAMs are implicated in a broad spectrum of processes ranging from inflammatory responses to airway epithelial repair. It has been proposed that their aberrant activity might lead to chronic inflammation and abnormal tissue remodeling in the lungs of COPD patients (Paulissen et al. 2009).

One of the ADAMs, a ubiquitously expressed Zn^2+^‐dependent disintegrin and metalloprotease 17 (ADAM17), formerly known as TNF*α* converting enzyme (TACE), is recognized as an important regulator of pulmonary inflammation, cell proliferation, and epithelial barrier function (Gooz 2010; Lemjabbar‐Alaoui et al. 2011). In bronchial epithelial cells, ADAM17 modulates these processes by cleaving membrane‐bound cytokines (TNF*α*), several EGF receptor (EGFR) agonists (TGF‐*α*, amphiregulin, epiregulin, HB‐EGF), cytokine receptors (IL6R, TNF‐R), growth factor receptors (NOTCH receptors), and adhesion proteins (L‐selectin, ICAM‐1, E‐cadherin) (Gomez et al. 2005; Bell et al. 2007; Baumgart et al. 2010; Gooz et al. 2012). Moreover, ADAM17 phosphorylation and activity is enhanced in airway epithelial cell lines and in undifferentiated primary cells upon exposure to cigarette smoke extract (Lemjabbar et al. 2003; Shao 2004; Lemjabbar‐Alaoui et al. 2011).

Our studies focus on two ADAM17 substrates implicated in COPD pathogenesis: the IL‐6 cytokine receptor (IL6R) and the growth factor amphiregulin (AREG), one of the EGFR agonists produced by bronchial epithelial cells (Richter et al. 2002). Elevated levels of IL6R have been observed in peripheral blood leukocytes of COPD patients (Edmiston et al. 2010), and recently genetic variants of IL6R have been linked with COPD severity (Pérez‐Rubio et al. 2016). However, the regulation of shedding of IL6R and AREG from COPD airway epithelium has not been studied. Upon shedding from epithelial cells, IL6R and AREG activate the shared interleukin receptor gp130 and EGFR, respectively, on epithelial cells (autocrine), as well as on underlying myofibroblasts and myeloid cells (paracrine) (Burgel and Nadel 2008; Nechemia‐Arbely et al. 2008; Kasina et al. 2009; Rose‐John 2012). Both IL6/IL6R/gp130 and AREG/EGFR/ERK pathways are involved in the resolution of lung inflammation and repair of injury, but also in progression of subepithelial fibrosis and collagen deposition (Zhou et al. 2012). These signaling pathways involve the JAK kinase and/or MAP kinase pathway, which are druggable targets in COPD pathology (Barnes 2013). Excessive ligand‐mediated EGFR activation results in epithelial hyperproliferation and increased production of the inducible mucin MUC5AC, processes observed in smokers with or without COPD (Lemjabbar et al. 2003; Shao 2004; Deshmukh et al. 2005; Kasina et al. 2009; Lemjabbar‐Alaoui et al. 2011; Li et al. 2011; Zhang et al. 2014). Moreover, EGFR activation results in subsequent transcriptional regulation of inflammatory mediators such as IL‐8 (Richter et al. 2002), a chemokine that has been implicated in COPD development.

So far, studies on the effect of cigarette smoke on epithelial ADAMs activity has largely relied on the use of airway epithelial cell lines or undifferentiated primary cell cultures, stimulated with an aqueous extract of cigarette smoke. However, whole cigarette smoke exposure and primary differentiated airway cell cultures represent more relevant physiological conditions. Firstly, fresh whole cigarette smoke (CS) contains unstable active components and particulate matter that are largely absent from extracts. Furthermore, immortalized epithelial cells are poor models of bronchial epithelium in situ, since they are frequently karyotypically unstable and heterogeneous, do not show characteristic features of differentiation and inherently carry multiple mutations in pathways essential for growth, differentiation, cell‐cell interaction, and polarization. Furthermore, submerged cultures of primary airway epithelial cells fail to differentiate. Finally, using cell lines does not allow a comparison of patient populations. Therefore, we examined the effect of whole CS exposure on shedding of the soluble interleukin‐6 receptor (sIL6R) and the EGFR‐ligand amphiregulin (AREG) by well‐differentiated, air–liquid interface cultured human primary bronchial epithelial cells (ALI‐PBEC).

This allowed us to compare CS‐induced ADAM17‐mediated protein shedding and mRNA expression of sIL6R and AREG in well‐differentiated ALI‐PBEC from COPD and non‐COPD (ex)smokers. Moreover, we established in this model the involvement of both EGFR and ADAM17 not only in shedding of ADAM17 substrates, but also in the regulation of mRNA levels of ADAM17 substrates and IL‐8. Finally, for the first time, we observed intracellular CS‐induced phosphorylated ADAM17‐substrate interaction via an in situ proximity ligation assay. Overall, our results provide novel insights into the activation of airway epithelial cells by cigarette smoke in COPD, and highlight a possible role of ADAMs and EGFR in COPD pathology.

## Materials and Methods

### Air–liquid interface cell culture of human primary bronchial epithelial cells

Human airway epithelial cells were obtained from macroscopically normal, anonymous bronchial tissue obtained from lung cancer patients undergoing resection surgery for lung cancer at LUMC. This material was be used for research according to the “Code of Conduct for Responsible Use” (FEDERA code) based on the condition that the patient has no objection against such use. Primary bronchial epithelial cells (PBEC) were isolated from tumor‐free lung resection material (Wetering et al. 2000), and passage 2 expanded cells were cultured at the air–liquid interface (ALI) to achieve mucociliary differentiation as previously described (Amatngalim et al. 2015). Briefly, 40,000 cells were seeded on 0.65 cm Transwell inserts (Corning Costar, Cambridge, MA) with a 0.4 *μ*m pore size, which were coated with 30 *μ*g/mL PureCol (Advanced BioMatrix, San Diego, CA), 10 *μ*g/mL Bovine serum albumin (Sigma‐Aldrich, St. Louis, MO), and 10 *μ*g/mL Fibronectin (isolated from plasma). Cells were cultured in Bronchial epithelial growth medium (BEGM) (Lonza, Verviers, Belgium) and Dulbecco's modified Eagle's medium (DMEM) (Gibco, Bleiswijk, The Netherlands) (1:1 mixture) containing 1 mmol/L Hepes (Lonza) and supplemented with SingleQuot supplements and growth factors according to the manufacturer's instructions (bovine pituitary extract, hydrocortisone, human epidermal growth factor, epinephrine, transferrin, insulin, T3 and retinoic acid; all from Lonza), additional 15 ng/mL retinoic acid (Sigma–Aldrich), 1 mg/mL BSA (Sigma–Aldrich), 100 U/mL penicillin, and 100 *μ*g/mL streptomycin (Lonza). PBEC were initially cultured on inserts in submerged conditions until cell layers were confluent. Next, apical medium was removed and cells were cultured at air‐exposed conditions for at least 2 weeks to allow mucociliary differentiation. Clinical history and lung function data were obtained from anonymized patients (Table 1), and COPD disease status was based on lung function data according to the Global Initiative for Chronic Obstructive Lung Disease (GOLD) classification (Vestbo et al. 2013). Donor cells were randomly allocated to experimental groups.

**Table 1:** COPD and non‐COPD patient characteristics

	COPD	non‐COPD	*P*‐value
Number of donors	15	11	
Gender (females/males)	4/11	2/9	
Age, years	70 ± 8	66 ± 6	0.1667
FEV_1_, % predicted	65 ± 16	81 ± 16	<0.01
FEV_1_/FVC %	55 ± 9	79 ± 9	<0.0001

Characteristics of PBEC donors. Age in years, and lung function as FEV_1_ (% predicted) and FEV_1_/FVC are shown as means ± SD. The mean differences were compared using the nonparametric Mann–Whitney test. COPD, chronic obstructive pulmonary disease, FEV1, Forced expiratory volume in one‐second, FVC, forced vital capacity.

### Cigarette smoke exposure

Air–liquid interface cultured human primary bronchial epithelial cells were exposed to whole cigarette smoke (CS) in an exposure model, adapted from (Beisswenger et al. 2004) and previously described in more detail (Amatngalim et al. 2015). In this model, ALI‐PBEC cultures were placed in either a CS‐ or air (negative control) exposure chamber located in a tissue incubator at 37°C and 5% CO_2_. Smoke derived from one cigarette (3R4F reference cigarettes [University of Kentucky, Lexington, KY]) was infused into the exposure chamber by a mechanical pump with a constant flow of 1 L/min, and equally distributed by a ventilator inside the chamber. After infusion (approximately 4–5 min), residual smoke was removed by infusion of air from the tissue incubator for 10 min. Directly after CS exposure, the basal medium of the cell cultures was refreshed and cells were incubated for the indicated periods of time. Untreated cells used as controls were subjected to the same procedure omitting the smoke (AIR).

### Inhibitors

TMI‐2 (1 μmol/L; PF‐5480090), a highly selective inhibitor of ADAM17 activity (Zhang et al. 2004), was obtained from Wyeth inc. (Philadelphia, Pennsylvania) and the selective EGFR inhibitor AG1478 (1 μmol/L) was from Sigma Aldrich. Cells were preincubated for 1 h with inhibitors before CS exposure, and directly after CS exposure media were replaced and inhibitors were freshly added.

### ELISA

ALI‐PBEC conditioned culture media were collected from the basolateral side of the inserts (1 mL), and apical washes were obtained by washing the apical surface with 100 *μ*L PBS at different time points, dependent on the experiment. Collected samples were diluted 1:1 with BEGM media and analyzed for IL6R and AREG by human IL6R or AREG ELISA kit, R&D. Further steps were performed according to the manufacturer's protocol. Data were corrected for the dilution factor and insert size, and the amount of the shed IL6R and AREG was expressed as pg/mL per cm^2^.

### RNA isolation and quantitative real‐time PCR

RNA was isolated using the miRNeasy Mini Kit (Qiagen) according to the manufacturer's instructions, and cDNA was synthesized by reverse‐transcription PCR using oligo(dT) primers (Qiagen) and Moloney murine leukemia virus (M‐MLV) polymerase (Promega, Leiden, The Netherlands). mRNA expression was determined by quantitative real‐time PCR as described previously (Amatngalim et al. 2015) with primer pairs presented in Table 2. mRNA expression was quantified using the standard curve method (Larionov et al. 2005), in which arbitrary expression levels were normalized to the housekeeping genes RPL13A and ATP5B. The housekeeping genes were selected based on stable expression using the “Genorm method” (Vandesompele et al. 2002).

**Table 2:** Quantitative‐PCR primers

Primer name		[ref]
IL8	FW 5′‐CAGCCTTCCTGATTTCTG‐3′	Amatngalim et al. (2015)
	RV 5′‐CACTTCTCCACAACCCTCTGC‐3′	
AREG	FW 5′GTGGTGCTGTCGCTCTTGATA 3′	Clarke et al. (2013)
	REV 5′ACTCACAGGGGAAATCTCACT3′	
IL6R full‐length form (full‐IL6R)	FW 5′GCTGTGCTCTTGGTGAGGAAGTTT3′	Rath et al. (2010)
	REV 5′CTGAGCTCAAACCGTAGTCTGTAGAAA3′	
IL6R alternatively spliced variant (spliced IL6R)	FW 5′GCGACAAGCCTCCCAGGTT3′	Rath et al. (2010)
	REV 5′CCGCAGCTTCCACGTCTTCTT3′	

FW, Forward primer; REV, Reversed primer.

### Proximity ligation assay

Chronic obstructive pulmonary disease ALI‐PBEC werefixed with 4% paraformaldehyde and permeabilized with 0.5% Triton‐X100 in PBS twice for 15 min, blocked with 1% BSA and 0.15% glycine. Next, they were incubated overnight in 4°C with two different first antibodies simultaneously: against ADAM17 (rabbit polyclonal, C‐terminal ADAM17, 25 *μ*g/mL, ab78162, Abcam) or ADAM17P^T735^ (rabbit polyclonal, phospho‐ADAM17 in position T735, 25 *μ*g/mL, ab60996, Abcam) and IL6R (goat anti‐human IL6R recognizing extracellular domain, 20 *μ*g/mL, AF‐227‐NA, R&D) or AREG (polyclonal goat anti‐human Areg, 25 *μ*g/mL, AF‐262, R&D). All washing steps were repeated three times with 0.5% Triton‐X100 in PBS (Sigma‐Aldrich). Further steps were performed according to the DuoLink manufacturer's protocol. Briefly, Proximity ligation assay (PLA) probes for anti‐goat PLUS and anti‐rabbit MINUS were incubated at 37°C for 1 h. Ligation and amplification steps were performed with Detection Red Reagent. Finally, inserts with ALI‐PBEC were cut out and mounted on the slides with DAPI (Vectashield mounting medium for fluorescence with DAPI, H‐1200). *z*‐stacks were acquired using confocal microscopy (Leica604).

### PLA image analysis

The number of dots was counted in the whole *z*‐stack with Image J software. The threshold values were adjusted with the *Intermodes* algorithm (the filter size set between 10 and 437 microns to exclude the small and large dots, which were in the range of 10% of the total dot count). The objects on the edges of the culture inserts were excluded from the analysis. The number of nuclei was counted in each *z*‐stack by hand to express the number of dots per nucleus.

### Statistical analysis

Data were analyzed with GraphPad Prism Software, using the appropriate statistical test, as indicated underneath each figure. Cells from various donors (number indicated by n in the legends of figures) were used for each experiment, samples were collected from two or three wells from a single donor, averaged and represented as a single dot in the figure. Statistical analysis was performed on the averaged data. Values are presented as mean with SEM values. Differences at *P*‐values <0.05 were considered to be statistically significant. ns > 0.05, *P < 0.05, **P < 0.01, **P < 0.001, ****P < 0.0001.

## Results

### CS induces shedding of IL6R and AREG by ALI‐PBEC into basolateral medium, but not apical

**Figure 1:**
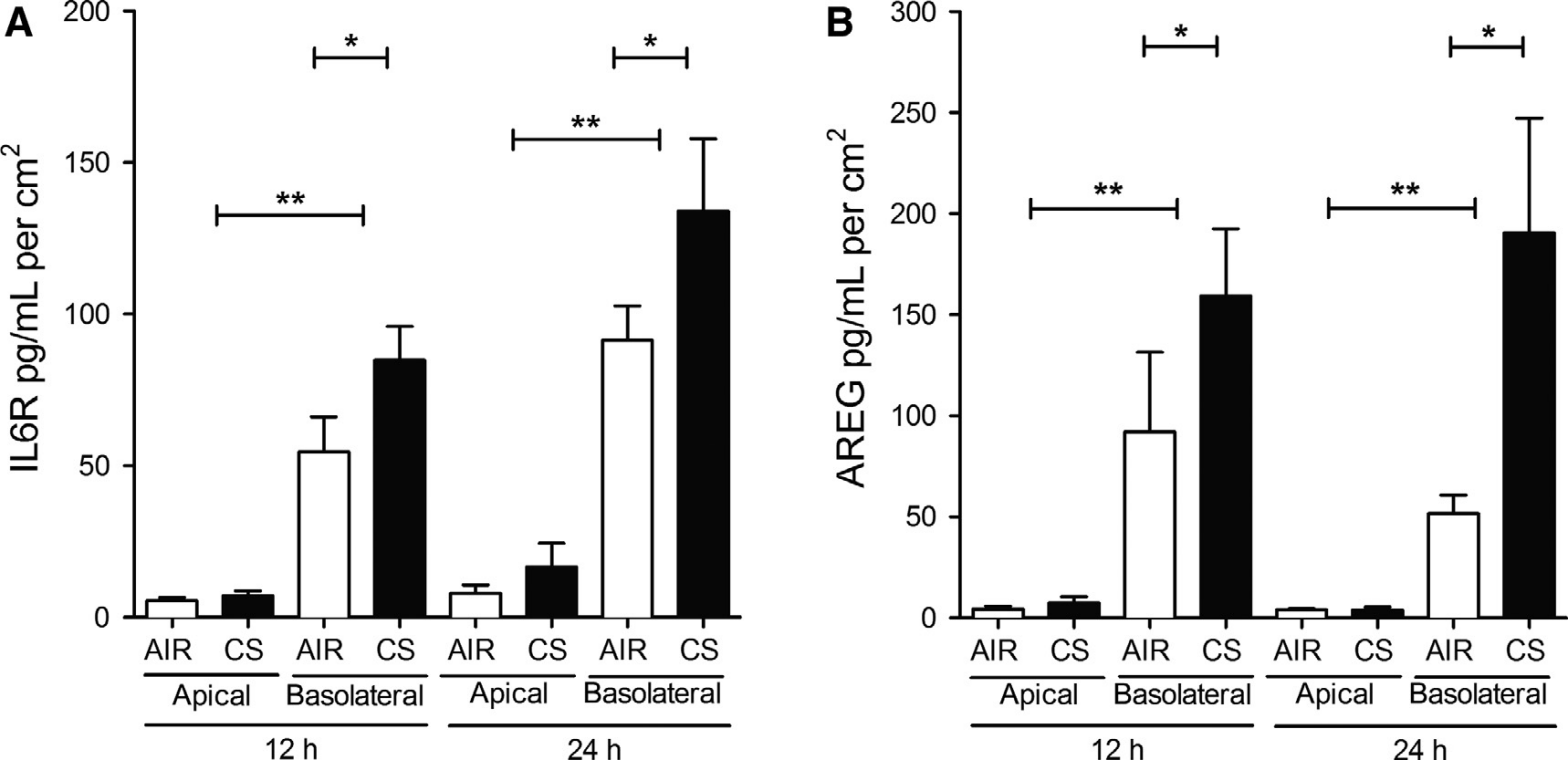
Cigarette smoke induces shedding of IL6R and AREG by ALI‐PBEC into basolateral medium. IL6R (A) and AREG (B) were mainly shed to the basolateral compartment in ALI‐PBEC (COPD donors at different stages, *n* = 12 for IL6R and *n* = 5 for AREG). Basolateral media (basolateral) were collected and apical PBS washes (apical) were performed 12 h and 24 h after CS or air exposure. Both IL6R and AREG were readily detectable in the basolateral compartment, and barely present in the apical washes. The response of cells from each donor was analyzed within one experiment using duplicate or triplicate inserts. Statistical analysis was performed by two‐way ANOVA (Bonferroni) on the averaged data from each donor, comparing apical versus basolateral shedding at air and CS exposure, and basolateral shedding at air versus CS exposure.

We first examined the effect of cigarette smoke (CS) exposure on the release of sIL6R and AREG by ALI‐PBEC at the apical surface and in the basal medium, which contains a maintenance level of EGF, associated with a basal level of EGFR activity. This was done, using a previously described whole CS exposure model (Amatngalim et al. 2015), in which CS caused a transient disruption in the airway epithelial barrier integrity, accompanied by minor cytotoxic effects measured at the apical surface. Both sIL6R and AREG were barely detectible in the apical washes collected from ALI‐PBEC of 17 COPD donors at different stages of disease, following exposure to either CS or air (Fig. 1A and B). In contrast, sIL6R and AREG were markedly released into the basal medium in both conditions. CS significantly increased release of sIL6R into the basal medium at 12 h postexposure, while AREG levels were increased at 12 and 24 h after CS exposure. These results demonstrate that shedding of sIL6R and AREG by ALI‐PBEC occurs mainly to the basolateral compartment, and is enhanced by CS exposure.

### CS significantly induces shedding of IL6R and AREG in COPD ALI‐PBEC but not in non‐COPD ALI‐PBEC

**Figure 2:**
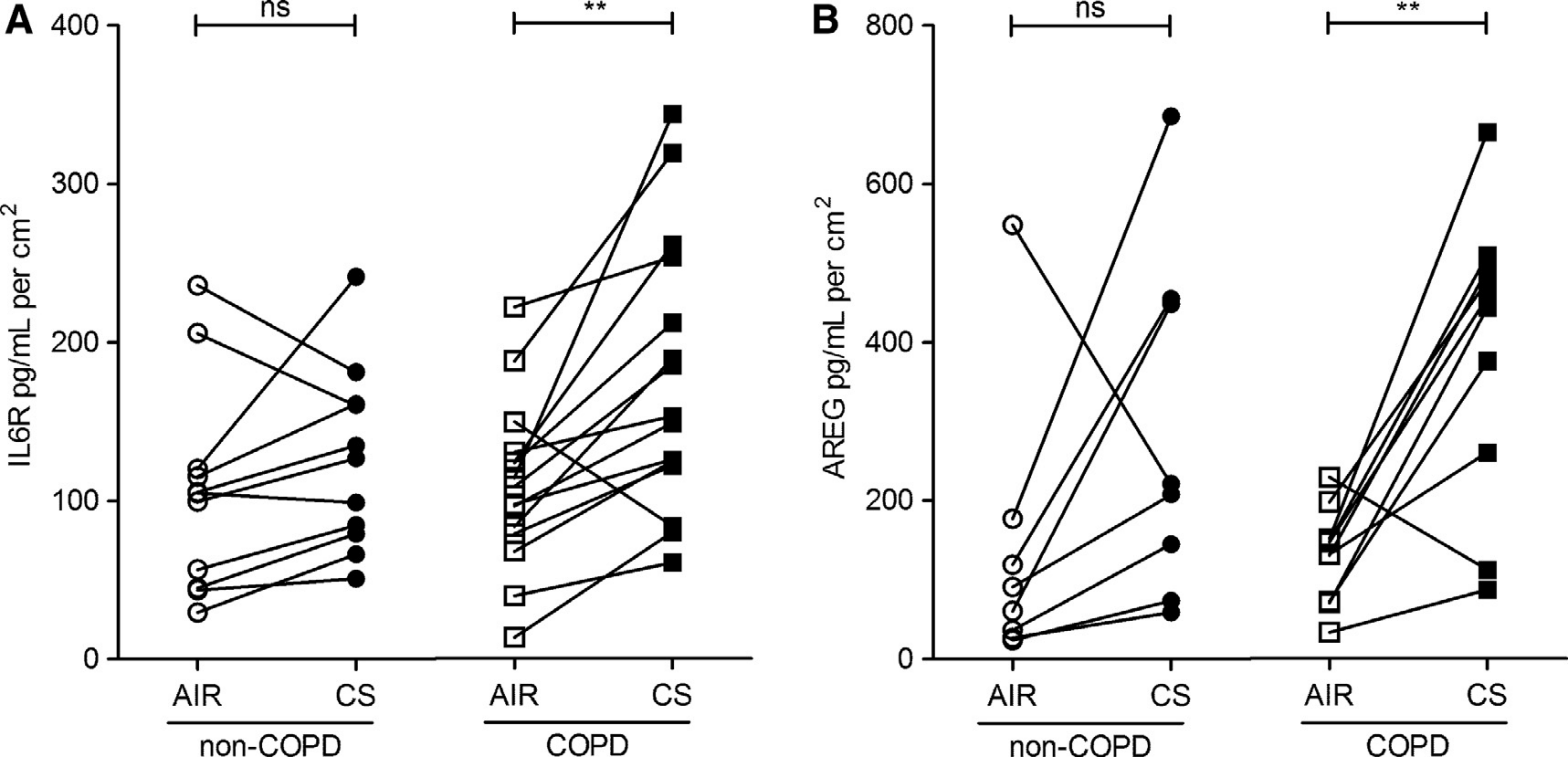
Cigarette smoke significantly induces shedding of IL6R and AREG into basolateral medium by COPD ALI‐PBEC. Soluble forms of IL6R (A) and AREG (B) shed into the basolateral compartment were detected 24 h after CS or air exposure in ALI‐PBEC derived from non‐COPD and COPD donors (Table 1). (A) IL6R levels were significantly increased 24 h after CS treatment in COPD‐ALI‐PBEC (*n* = 15), but this increase was not significant in the non‐COPD group (*n* = 11 donors). (B) Similarly, CS exposure significantly increased AREG levels in ALI‐PBEC cells derived from COPD donors (*n* = 10), but not in non‐COPD ALI‐PBEC (*n* = 8 donors). Statistical analysis: paired *t*‐test. *n* refers to the number of donors, duplicate or triplicate data were averaged per donor. Statistical analysis was performed on the averaged data from each donor.

Next, we explored whether shedding of sIL6R and AREG differs between ALI‐PBEC isolated from COPD patients and non‐COPD (ex)‐smokers upon CS and air exposure. Based on the previous result, the release was only determined in the basal medium 24 h after exposure. Shedding of sIL6R and AREG did not differ between COPD and non‐COPD ALI‐PBEC exposed to air (Fig. 2A and B), indicating no differences at baseline conditions. In contrast, shedding of sIL6R (Fig. 2A) and AREG (Fig. 2B) was significantly higher after CS exposure only in COPD ALI‐PBEC, and not in non‐COPD ALI‐PBEC. These data show that CS‐induced release of sIL6R and AREG was more pronounced in airway epithelial cells from COPD in comparison to non‐COPD donors.

### CS‐induced IL6R and AREG mRNA expression is lower in COPD ALI‐PBEC compared to non‐COPD cultures

We further determined mRNA expression of IL6R and AREG in CS and air exposed ALI‐PBEC cultures from COPD and non‐COPD patients. The soluble form of IL6R can be generated either by shedding of the membrane anchored form or by de novo synthesis of the alternatively spliced isoform that differs at the C‐terminus (Rose‐John 2012). Therefore, we determined mRNA expression levels of both IL6R variants: the membrane‐anchored (full‐IL6R mRNA) and the alternatively spliced (spliced‐IL6R mRNA) variant.

**Figure 3:**
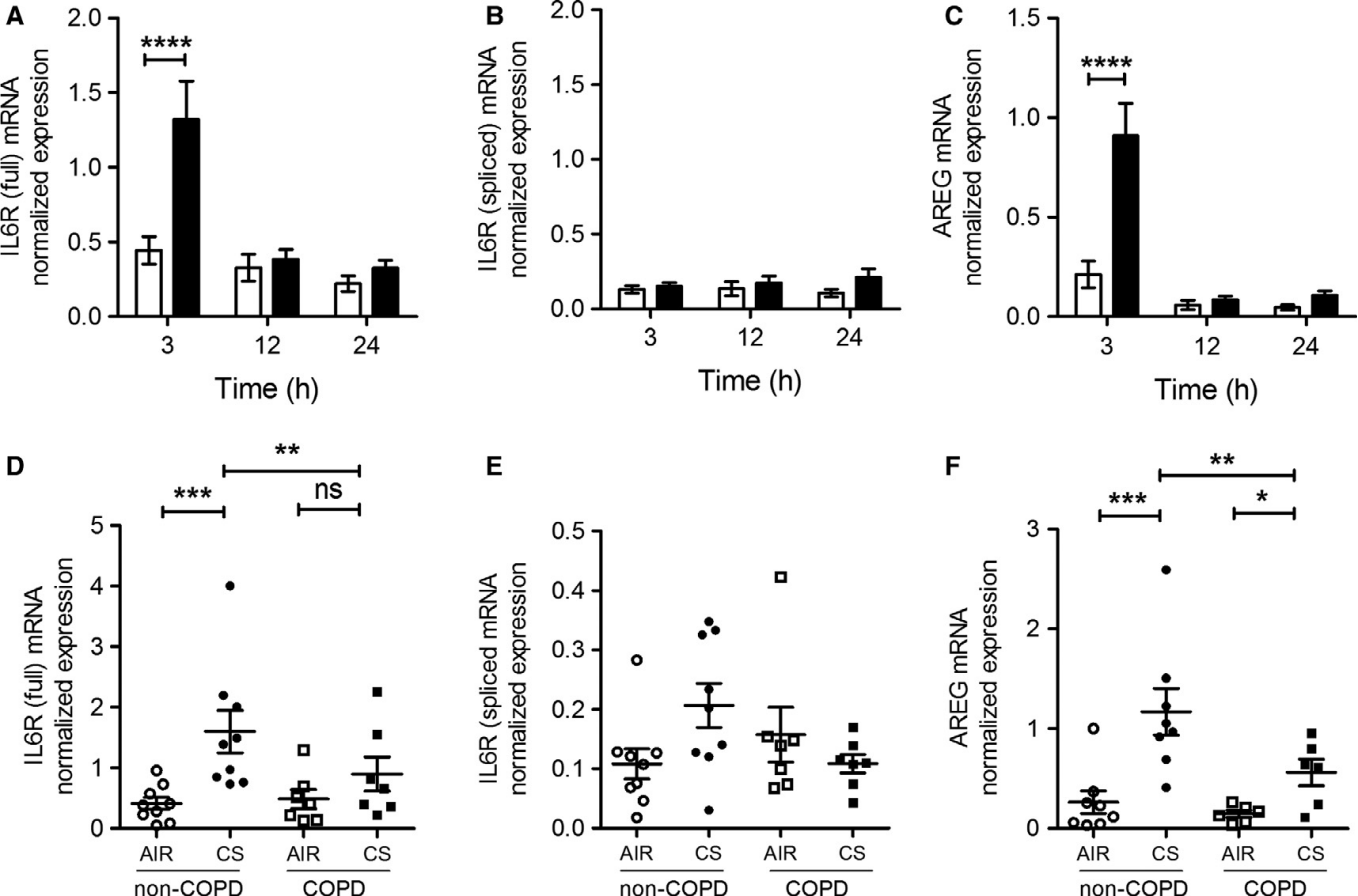
CS exposure transiently enhances IL6R and AREG mRNA expression in COPD and non‐COPD ALI‐PBEC. mRNA levels of the IL6R full‐length variant (full‐IL6R) (A), the IL6R splice variant (spliced‐IL6R) (B) and AREG (C) were determined by qPCR 3, 12, and 24 h after CS (black bars) or air exposure (open bars) (*n* = 14 unspecified donors). A transient induction of full‐IL6R (A) and AREG (C), but not spliced‐IL6R (B) was observed at 3 h after CS exposure. In COPD (*n* = 7) and non‐COPD (*n* = 8) ALI‐PBEC, mRNA of full‐IL6R (D), spliced‐IL6R (E), and AREG (F) were determined 3 h after CS exposure. mRNA expression of full‐IL6R and AREG, was lower on average but not statistically significant in COPD compared to non‐COPD donors. Data were normalized for expression against two reference genes (ATP5B and RPL13A). *n* refers to the number of donors. The response of cells from each donor was analyzed within one experiment using duplicate inserts and data were averaged per donor. Statistical analysis was performed on the averaged data from each donor. Statistical analysis: Two‐way ANOVA With Tukey's multiple comparison test.

Time‐course analysis revealed that CS increased full‐IL6R mRNA 3 h after exposure, but not at later time points (Fig. 3A). In contrast, baseline expression of spliced‐IL6R mRNA did not differ from the expression after CS treatment (Fig. 3B), suggesting that the increase in sIL6R protein levels in culture supernatants did not result from alternative splicing. Similar to full‐IL6R mRNA, CS significantly induced AREG mRNA expression 3 h after exposure, but not at later time points (Fig. 3C). These findings suggest that the CS‐induced increase in IL6R and AREG shedding is mediated at least in part *via* regulation of their mRNA expression levels.

Baseline expression of full‐IL6R and AREG mRNA did not differ between COPD and non‐COPD ALI‐PBEC (Fig. 3D and F). After CS exposure, full‐IL6R and AREG mRNA were expressed at higher levels in both non‐COPD and COPD ALI‐PBEC. Interestingly, after CS induction, COPD cells expressed full‐ILR and AREG at lower levels on average but this did not reach statistical significance (Fig. 3D and F). Spliced‐IL6R mRNA expression did not differ between investigated groups either after CS or air exposure (Fig. 3E). These findings suggest that COPD patients may have impaired transcriptional or posttranscriptional responses to inflammatory and tissue regenerative triggers. The apparent contrast with the more pronounced shedding from COPD cells after CS challenge (Fig. 2) suggests that posttranslational mechanisms determine shedding rate, rather than substrate mRNA levels.

### ADAM17 is required for CS‐induced release of IL6R and AREG in ALI‐PBEC

**Figure 4:**
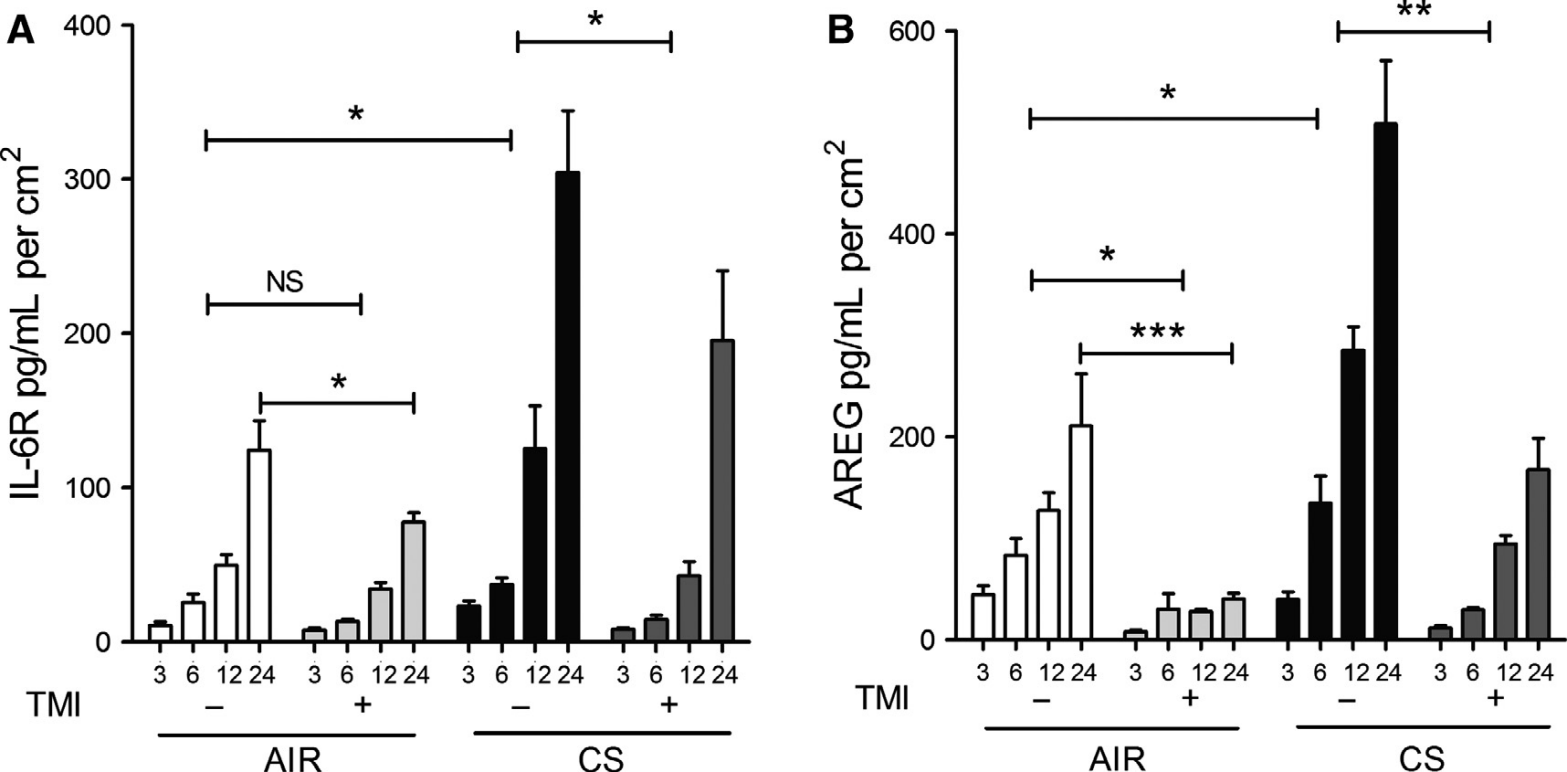
ADAM17 is involved in the release of soluble IL6R and AREG in ALI‐PBEC. The selective ADAM17 inhibitor, TMI‐2 (Zhang et al. 2004) decreases basal and CS‐induced IL6R (A,B) and AREG (C,D) shedding in ALI‐PBEC cells (*n* = 3 COPD donors) at 3, 6 (A,C), 12, and 24 h time points (B,D). *n* refers to the number of donors. The response of cells from each donor was analyzed within one experiment using duplicate or triplicate inserts and data were averaged per donor. Statistical analysis was performed on the averaged data from each donor, by two‐way ANOVA (Bonferroni), confirming first the effect of CS on IL6R and AREG shedding at different time points, and second the effect of TMI‐2 on shedding during air and CS exposure.

To confirm the previously established involvement of ADAM17 in the shedding process of IL6R and AREG in our model, we used the selective ADAM17 inhibitor TMI‐2 (Wyeth) (Zhang et al. 2004). TMI‐2 only partially decreased baseline IL6R release at all investigated time points (Fig. 4A), plausibly because release of the product of spliced‐IL6R mRNA, which cannot be distinguished from shed IL‐6R with the available antibodies, is not sensitive to inhibitors of ADAMs (Vermes et al. 2012). In contrast, TMI‐2 significantly decreased baseline AREG shedding at all time points (Fig. 4B). Importantly, CS‐induced shedding of IL6R and AREG was significantly inhibited by TMI‐2 at all time points after CS exposure, indicating that ADAM17 activity is involved in CS‐induced ADAM17 substrate release (Fig. 4).

### ADAM17‐ and ADAM17P‐substrate interactions are increased after CS exposure in an intracellular compartment of ALI‐PBEC

**Figure 5:**
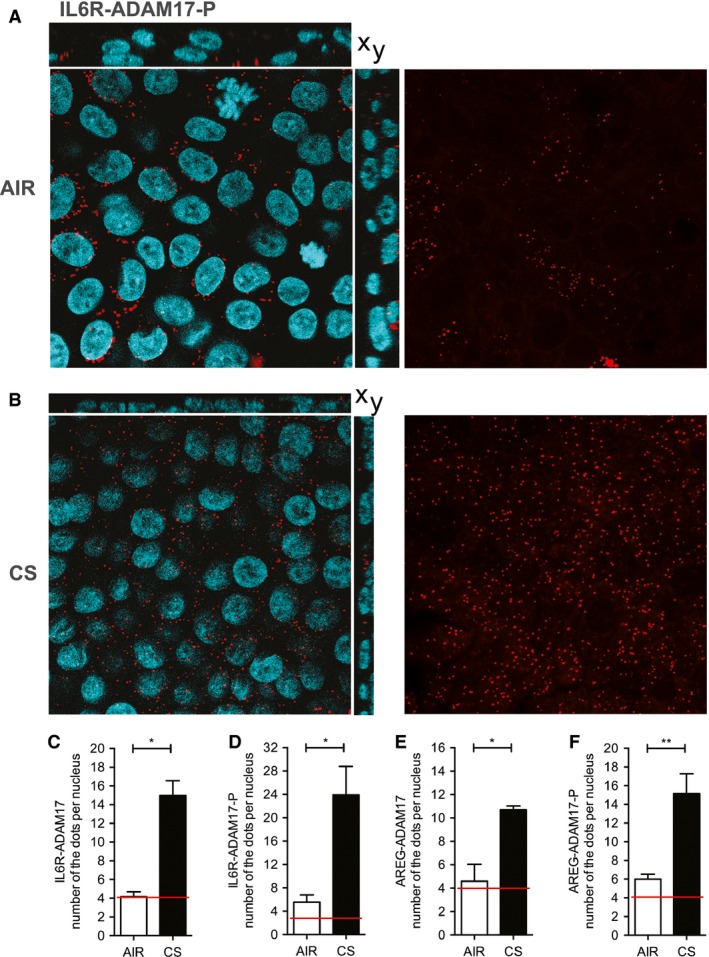
CS increases substrate‐ADAM17 and substrate‐ADAM17‐P^T^ ^735^ interactions in pseudostratified COPD ALI‐PBEC cells. The proximity ligation assay (PLA) signal in COPD‐ ALI‐PBEC exposed to CS or air was performed for the following interactions: IL6R‐ADAM17, IL6R‐ADAM17P^T^ ^735^, AREG‐ADAM17, and AREG‐ADAM17‐PT ^735^. Relevant control data are shown in Figure S1. Here, we show a representative figure of IL6R‐ADAM17P^T^ ^735^ 3 h after air (A) and CS exposure (B). Left panels show merged signals of nuclei (blue) and PLA (red) in the *x*–*y* sections of the confocal *z*‐stack and right panel presents PLA signal in the apical region (red dots). The number of PLA interactions was counted for all interactions as described in the methods section and expressed per nucleus 3 h after CS or air exposure in the whole *z*‐stack of the ALI‐PBEC (C–F). The red lines on the graphs indicate the maximal dot count in the PLA assay controls, in which one of the antibodies for the interaction was omitted (background staining not shown). For each interaction, cells from one donor were analyzed. Different filters (*n* = 4) were used to show distinct interactions. Statistical analysis: unpaired *t*‐test.

Next, we explored the interactions of IL6R or AREG with ADAM17 3 h after CS treatment in ALI‐PBEC with an in situ proximity ligation assay (PLA) (Fredriksson et al. 2002), using antibodies against ADAM17 phosphorylated at Thr735 (ADAM17‐P^T735^) or total ADAM17. Protein IL6R/AREG‐ADAM17 and IL6R/AREG‐ADAM17‐P^T735^ interactions were visualized as fluorescent red dots in x‐y confocal sections (representative confocal pictures shown in Fig. 5A and B). In air‐exposed cells, PLA signals were largely confined to the basal region, as in the control incubations, and not significantly higher than background (data not shown), as indicated by red lines in Figure 5C–F (relevant control data are shown in Figure S1). Interestingly, CS exposure significantly increased the total number of PLA signals for interactions of IL6R or AREG with ADAM17 (Fig. 5C and E). We observed that CS strongly enhanced interactions of IL6R or AREG with ADAM17‐P^T735^ (Fig. 5D and F), which further extends previous findings showing that ADAM17 is phosphorylated after smoke extract (CSE) exposure in submerged immortalized NCI‐H292 cells (Lemjabbar‐Alaoui et al. 2011). CS‐induced PLA signals of substrate‐ADAM17 and substrate‐ADAM17‐P^T735^ were primarily detected in the apical region of the cells and were not confined to a lateral membrane pattern suggesting an intracellular vesicular localization of protein complexes in ALI‐PBEC. These data for the first time demonstrate that CS exposure strongly increases the interaction of ADAM17 and ADAM17‐P^T735^ with IL6R or AREG in an intracellular vesicular compartment of ALI‐PBEC, suggesting a CS induced effect on protein trafficking.

### EGFR is required for basal and induced AREG shedding in ALI‐PBEC

**Figure 6:**
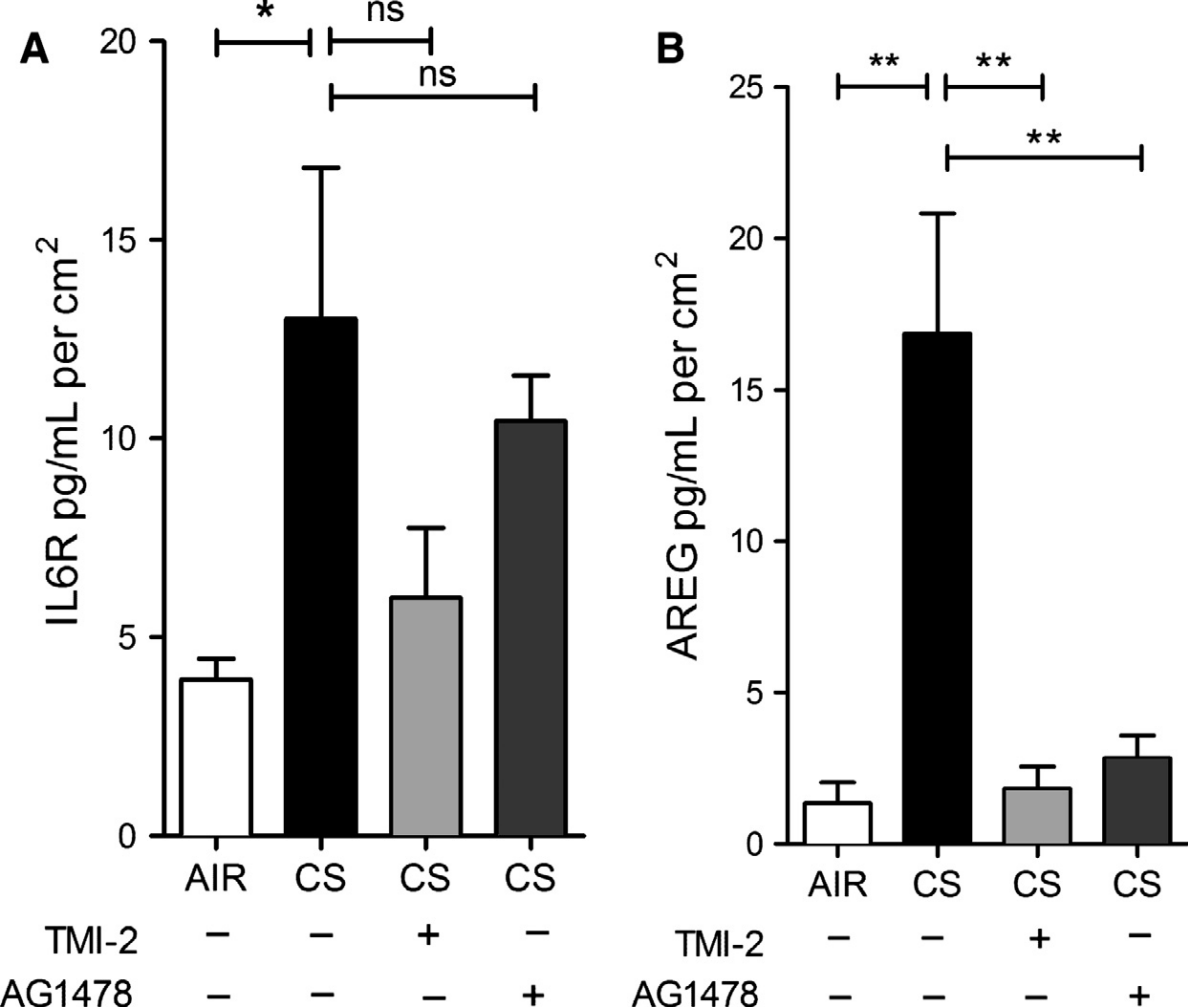
IL6R and AREG shedding depends on ADAM17 and EGFR activity in COPD‐ALI‐PBEC. COPD ALI‐PBEC (*n* = 3 donors) were starved for growth factors for 48 h prior to CS or air exposure. Three hours after CS exposure, IL6R (A) and AREG (B) shedding were significantly increased compared to air. The ADAM17 inhibitor (1 μmol/L TMI‐2) and the EGFR inhibitor (1 μmol/L AG1478) significantly reduced AREG, but IL6R shedding to a lesser extent. The response of cells from each donor was analyzed within one experiment using triplicate inserts and data were averaged per donor. Statistical analysis was performed on the averaged data from each donor by one way ANOVA (Tukey multiple comparison test), only relevant comparisons are shown, air versus CS‐treated cells and the effect of inhibitors in CS‐treated cells.

ADAM17‐dependent shedding of EGFR ligands such as AREG results in activation of EGFR through an autocrine feedback loop, which modulates basal EGFR activity (DeWitt et al. 2001). This mechanism was shown to be activated by CS extract in submerged cultured PBEC and in cell lines (Lemjabbar et al. 2003). In our experimental set‐up, we have previously shown that CS enhances basal EGFR activity by increasing its phosphorylation (Amatngalim et al. 2015). To illustrate the involvement of EGFR in CS‐induced ADAM17‐related shedding in ALI‐PBEC, we assessed sIL6R and AREG shedding after starvation for growth factors, using medium devoid of EGF and bovine pituitary extract (BPE). Removing these factors from the medium substantially reduced baseline shedding of IL6R and AREG (Fig. 6), when compared to standard culture conditions including EGF and BPE (Fig. 4A and C). Both sIL6R and AREG release were significantly increased at 3 h after CS exposure. The selective ADAM17 inhibitor TMI‐2 and the EGFR tyrosine kinase inhibitor (AG1478) added prior to CS exposure, partially inhibited sIL6R shedding, consistent with a substantial contribution of the ADAM‐insensitive splice variant sIL‐6R levels in the basal medium (Fig. 6A). AG1478 strongly impaired AREG shedding, to a similar extent as TMI‐2 (Fig. 6B). These findings together demonstrate a critical role of EGFR activation in ADAM17‐mediated basal and CS‐induced shedding activity.

### EGFR and ADAM17 are required for CS‐induced IL6R and AREG mRNA expression

**Figure 7:**
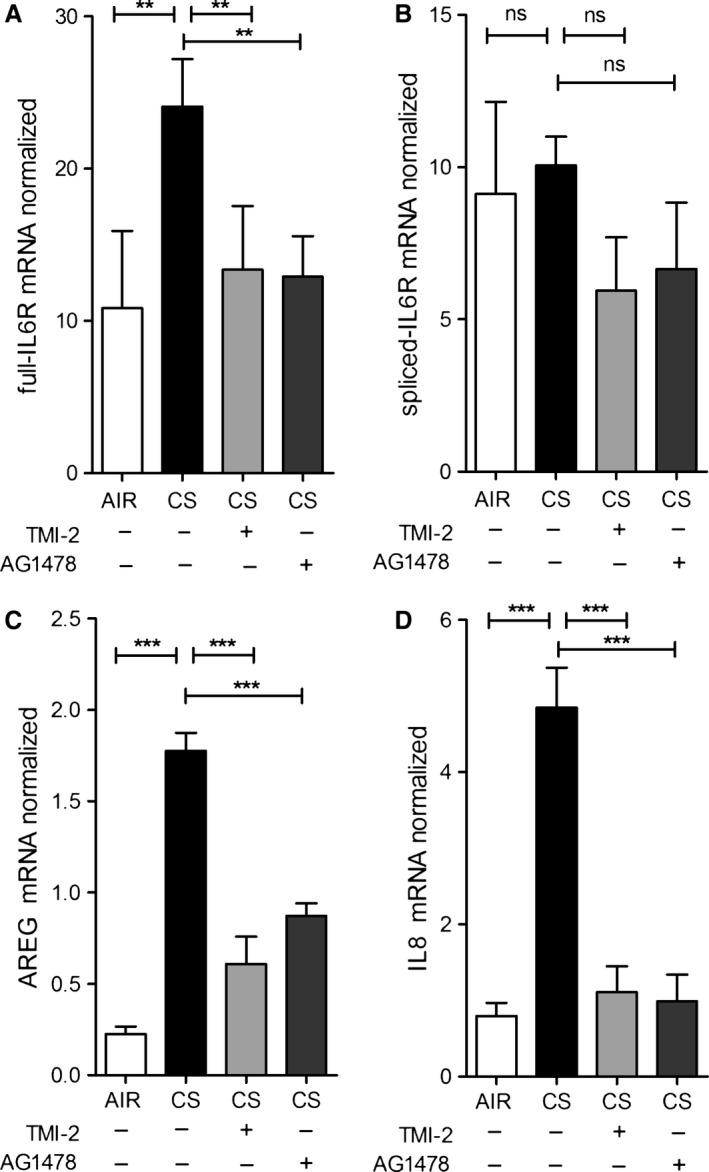
IL6R and AREG mRNA expression are regulated by ADAM17 and EGFR activity in ALI‐PBEC. ALI‐PBEC (*n* = 3 donors) were starved for growth factors for 48 h prior to CS exposure. At 3 h, CS‐induced mRNA levels of full‐length IL6R (A), AREG (C), and IL8 (D) were diminished upon ADAM17 (1 μmol/L TMI‐2) and EGFR (1 μmol/L AG1478) inhibition, whereas that of the alternatively spliced form of IL6R was not affected (B). *n* refers to the number of donors. The response of cells from each donor was analyzed within one experiment using triplicate inserts and data were averaged per donor. Statistical analysis was performed on the averaged data from each donor, by one‐way ANOVA (Tukey multiple comparison test), only relevant comparisons are shown, air versus CS‐treated cells and the effect of inhibitors in CS‐treated cells.

We previously observed that EGFR activation is involved in CS‐induced expression of several genes in ALI‐PBEC (Amatngalim et al. 2015). The molecular mechanism by which CS activates EGFR are not known. Here, we explored the effect of ADAM17 and EGFR inhibition on CS‐induced IL6R and AREG mRNA levels in ALI‐PBEC. At 3 hours after CS exposure in the absence of EGF in the medium, both TMI‐2 and AG1478 significantly impaired CS‐induced expression of full‐L6R mRNA (Fig. 7A), but not the splice variant (Fig. 7B). Both inhibitors strongly diminished CS‐induced AREG mRNA levels (Fig. 7C) as well as IL‐8 mRNA expression (Fig. 7D). Overall, these findings for the first time demonstrate that ADAM17, next to EGFR, is essential in the CS‐induced mechanism regulating not only the mRNA of ADAM17 substrates (IL6R and AREG), but also IL‐8 in ALI‐PBEC.

## Discussion

Many studies have demonstrated that airway epithelial cells are activated by exposure to environmental triggers like cigarette smoke, which contributes to COPD pathology (Rusznak et al. 2000; Schulz et al. 2004; Heijink et al. 2012; Amatngalim et al. 2015). In contrast to most studies, we used fresh whole cigarette smoke instead of (aged) cigarette smoke extract, and ALI‐differentiated PBEC from COPD and non‐COPD donors instead of submerged cultures of nondifferentiated primary cells or cell lines.

While the obvious advantage of this approach is that we can study well‐differentiated primary cells from different patient populations, a limitation is that confirmation of data obtained with experimental pharmaceutics by, for example, gene editing or RNAi technology is not feasible in this context. Aside from efficiency issues and off‐target effects in primary cells, knocking down EGFR or ADAM17 likely affects the growth and differentiation of primary bronchial epithelial cells, which essentially defeats our purpose. However, the two inhibitors that we apply here to inhibit EGFR (AG1478) and ADAM17 (TMI‐2), respectively are widely used and are known to be highly selective.

**Figure 8:**
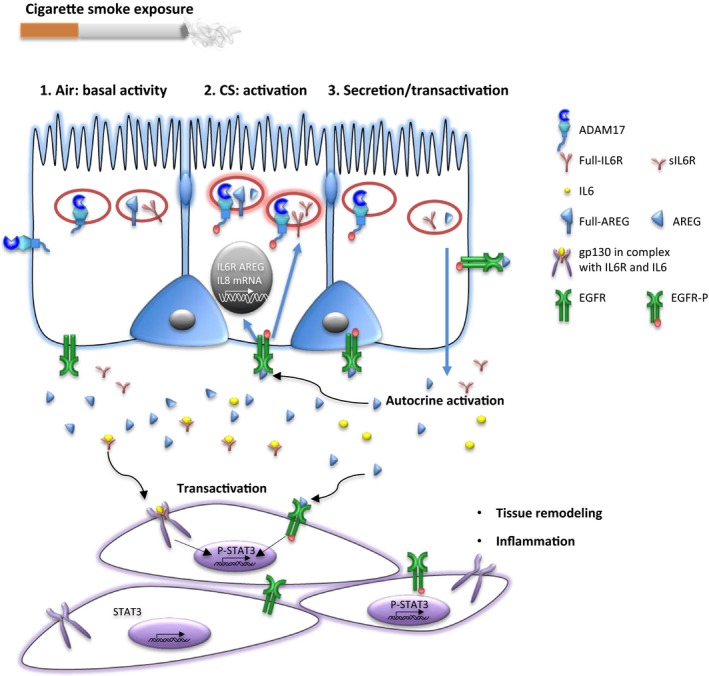
Cigarette smoke exposure activates EGFR‐ADAM17 axis in airway epithelial cells. Under basal conditions, there is ADAM17 related AREG and IL6R shedding activity, depending on the level of EGFR activity (compare Figs 4 and 6). Cigarette smoke exposure (CS) initiates an interaction of the phosphorylated form of ADAM17 (ADAM17‐P) with the full‐length transmembrane forms of IL6R and AREG in an intracellular compartment of the airway epithelial cell (Fig. 5), resulting in proteolysis and subsequent secretion (shedding) of the soluble active domains of IL6R and AREG toward the basolateral compartment. This involves both ADAM17 and EGFR activity (Figs 4 and 6). CS exposure also affects IL6R and AREG gene expression or mRNA stabilization in airway epithelial cells through ADAM17 and EGFR activation. sAREG and sIL6R secreted towards the basolateral compartment may change the level of activity of EGFR and the interleukin receptor IL6st/gp130 on the airway epithelial cells (autocrine). This may contribute to the activity of the EGFR/ADAM17 axis (positive feedback), which is likely kept in check by inactivation of internalized EGFR. Paracrine activity of sAREG and sIL6R may further transactivate EGFR and the interleukin receptor IL6st/gp130 on the underlying myofibroblasts, and myeloid cells, activating downstream pathways, including STAT3, involved in inflammation, collagen deposition, and myofibroblast proliferation.

Importantly, our data demonstrate for the first time that CS triggered increase of basal shedding of IL6R and AREG into the basal medium, in the presence of EGF in the growth medium providing basal EGFR activity, was more pronounced in ALI‐PBEC derived from COPD patients compared to non‐COPD controls. We further report the ability of CS to increase mRNA expression of these genes in an EGFR‐ and ADAM17‐dependent way in ALI‐PBEC cells, under these conditions, with a lower tendency to induction in the COPD group. These results extend previous studies showing dysregulated responses of COPD airway epithelial cells to cellular stress, and provide novel evidence for the mechanism of CS‐induced and COPD‐related proinflammatory and profibrotic responses (Fig. 8).

The differential effect of CS on sIL6R and AREG release between COPD and non‐COPD ALI‐PBEC might be related to differences in epithelial barrier function as previously described (Heijink et al. 2014). Using the current CS exposure system, we have previously shown that CS causes a transient decrease in epithelial barrier function (Amatngalim et al. 2015). However, in contrast to Heijink et al., we did not observe differences between COPD and non‐COPD cultures at baseline conditions and upon CS exposure (G.D. Amatngalim et al., unpubl. data), which may be explained by the fact that Heijink et al. focused on severe (GOLD stage IV) COPD. Another explanation might be differences in epithelial cell differentiation, as it has been shown that COPD epithelial cells display a more mesenchymal phenotype due to enhanced autocrine expression of TGF‐*β*1 (Gohy et al. 2015).

As previously shown, the EGFR‐ADAM17 pathway is essential for IL‐8 release from a bronchial epithelial cell line exposed to particulate air pollution (Ovrevik et al. 2011) and implicated in CS extract‐induced expression of the mucin MUC5AC (Shao 2004). Further, autocrine production of EGFR ligands is involved in CS‐induced IL‐8 release from airway epithelial cells (Richter et al. 2002). Our studies extend these observations by showing the involvement of the ADAM17‐EGFR pathway in the release of IL6R and AREG upon CS exposure of differentiated PBEC, both in the presence (Fig. 4), and absence (Fig. 6) of EGFR ligand (EGF) in the basal medium respectively. Notably, the basal shedding rates are considerably lower in cells preincubated in medium lacking EGF, resulting in a much larger ADAM17‐ and EGFR‐dependent stimulation effect of CS (compare Figs 4 and 6). Which of these extreme conditions of basal EGFR activation apply in normal and COPD lungs in situ, and to what extent autocrine feedback signaling through ADAM‐dependent EGFR ligand shedding determines EGFR activity (Fig. 8) remains to be established.

Additionally, EGFR and ADAM17 were both essential for CS‐induced IL6R and AREG mRNA expression (Fig. 7). These results provide novel insights into the mechanisms of airway epithelial cell activation by cigarette smoke in COPD, and highlight a role of ADAMs and EGFR in this process (Fig. 8).

We further found that CS increases shedding of IL6R and AREG to the basal medium, but not to the apical side (Fig. 1). This is in line with report in polarized Madin‐Darby canine kidney cells (MDCK cells) showing that newly synthetized AREG is directly delivered to the basolateral surface with >95% efficiency (Brown et al. 2001). However, this is in contrast to the secretion of the innate immune mediators IL‐8 and ribonuclease 7, which were also detected at the apical surface (Amatngalim et al. 2015). A polarized ADAM17‐mediated secretion toward underlying tissue may be relevant for lung tissue remodeling through autocrine, paracrine, extracrine (exosomal targeted receptor activation) pathways in COPD (Booth et al. 2007; Zhou et al. 2012). Further examination of this phenomenon in epithelial–mesenchymal co‐culture systems is in progress.

Amphiregulin release and phosphorylation of ADAM17 after CS extract treatment in ALI‐PBEC has been previously detected by ELISA or western Blotting (Lemjabbar‐Alaoui et al. 2011). Our proximity ligation assay (PLA) data show for the first time that CS‐induced shedding involves an intracellular interaction between phosphorylated ADAM17 and its substrates (Figs 5 and 8), whereas the majority of the literature suggests that shedding occurs mainly at the plasma membrane surface. This interaction likely takes place in intracellular membranes that sequester active phosphorylated ADAM17 and its transmembrane substrates upon activation. This process may relate to the transient change in barrier function upon CS treatment in our system and the subsequent activation of EGFR (Amatngalim et al. 2015). Our observation is supported by other reports showing the presence ADAM17 or its substrates in a vesicular compartment in lysosomes (Ebsen et al. 2015), endosomes (Gephart et al. 2011; Dombernowsky et al. 2015), and exosomes negative for the ER marker calreticulin (Higginbotham et al. 2011). Moreover, Gutwein et al. demonstrated that ADAM10‐mediated L1 migration factor cleavage occurs in Golgi‐derived vesicles in tumor cells (Gutwein et al. 2002). This was further supported by a recent paper suggesting that also ADAM10/ADAM17‐mediated release of soluble FasL occurs from an intracellular vesicular pool of secretory lysosomes in stimulated T lymphocytes (Ebsen et al. 2015).

Moreover, after ligand binding, EGFR traffics in endosomes from the plasma membrane to an intracellular compartment to continue its signaling (Vieira et al. 1996; Teis et al. 2006). EGF‐dependent MAPK signaling occurs from late endosomes and lysosomes (de Araujo et al. 2013). Interestingly, the MAPK/ERK pathway regulates trafficking of ADAM17 phosphorylated at Thr735 from the endoplasmic reticulum toward the plasma membrane (Soond 2005; Hilliard et al. 2011), which can be also activated through ligand binding to EGFR. Higginbotham et al. showed that AREG containing exosomes are rapidly internalized by recipient cells in an EGFR‐dependent manner (Higginbotham et al. 2011), enhancing invasion of LM2‐4175 cells through Matrigel and wound healing. In our ALI‐PBEC system, we observed a predominantly lateral localization of EGFR under basal culture conditions. After exposure to CS, we observed a more cytoplasmic localization, consistent with EGFR activation (Figure S2). Therefore, in line with these and published observations, our findings suggest that in HBEC‐ALI, CS triggers EGFR‐mediated trafficking of ADAM17 and its substrates to a common subcellular compartment to allow proteolysis and subsequent secretion of soluble products (Fig. 8). At this time, we cannot establish to what extent autocrine signaling through shed ADAM substrates determine this response, or whether alternative mechanisms such as transactivation by intracellular kinases or oxidation or the extracellular receptor domain plays a role. Additional studies of triggered trafficking of EGFR, ADAM17‐P, and its substrates in polarized airway cells are required to further establish this mechanism.

In addition to CS‐enhanced release through ADAM17 enzymatic activity, we observed transiently enhanced mRNA expression of both AREG and full‐IL6R in ALI‐PBEC upon CS exposure (Figure 3). CS did not affect the level of the alternatively spliced form of IL6R, so we conclude that alternative splicing unlikely contributes to CS‐enhanced release of IL6R. Previously, we observed upregulated IL‐8 mRNA expression in ALI‐PBEC exposed to CS as a result of enhanced EGFR phosphorylation and activation of the downstream MAPK/ERK1/2 signaling pathway (Amatngalim et al. 2015). Here, we report that CS‐induced IL6R and AREG mRNA expression was also reduced upon EGFR inhibition. In addition, we show here for the first time that inhibition of ADAM17 has the same effect on these mRNA levels (Fig. 7). Therefore, our data suggest that CS enhances factors common for activation of IL6R, AREG, and IL‐8 mRNA expression likely via an autocrine ADAM17‐EGFR axis (Fig. 8).

The transcriptional and posttranscriptional regulation of these genes upon inhaled toxic substances has not been fully elucidated. Induced EGFR signaling is able to activate transcription of target genes. In addition, it has been shown that CS extract enhances HuR‐mediated IL‐8 mRNA stability in airway epithelial cells (Hudy and Proud 2013). Moreover, UV‐exposure of keratinocytes enhances mRNA HuR‐mediated stability of AREG, in an EGFR‐dependent manner (Nakayama et al. 2013). These observations suggest that CS‐induced activation of EGFR enhances sIL6R, AREG, and IL8 mRNA stability in ALI‐PBEC. Furthermore, mRNA regulation may be altered in cultured airway epithelial cells from COPD patients (Steiling et al. 2013). CS‐treated COPD ALI‐PBEC expressed lower AREG and IL6R mRNA levels on average compared to non‐COPD controls, but this difference was not statistically different (Fig. 3). Further studies on mRNA stability in this system are required to establish this. Nevertheless, this observation contrasts with the shedding data (Fig. 2) and suggests that ADAM17‐dependent AREG and sIL6R output is not primarily regulated on the mRNA level, but involves posttranslational regulation.

Our data support the relevance of the ADAM17/EGFR pathway in COPD development and progression. Selective inhibitors of ADAM17, EGFR, and other components of this signaling pathway such as JAK and MAPK potentially expand therapeutic possibilities. The development of ADAM inhibitors for clinical use has been studied intensively (Moss et al. 2008; Duffy et al. 2011; Dreymueller et al. 2015). In cellular and animal tumor models, positive results were recorded (Witters et al. 2008). An ADAM17 inhibitor, TAPI‐0, reduced bleomycin‐induced lung inflammation (Lee et al. 2012). The selective inhibitor TMI‐2 used in this study, reduced LPS‐induced inflammation in vivo (Zhang et al. 2004). However, due to a lack of target specificity of available compounds, and side effects associated with the various other biological functions of ADAMs, chronic and systemic application of these compounds in humans is so far prohibited (Arribas and Esselens 2009). Clearly, more advanced intervention tools are required. Our data offer new insights in the regulation of mRNA expression, secretion, and release of ADAM17 substrates in airway epithelial cells upon triggering, which in combination with state of the art molecular design and advanced organotypic cellular modeling of airways could allow development of more selective inhibitors, targeted to specific cells and subcellular domains.

In summary**,** this study provides evidence that ADAM17‐mediated release and shedding of IL‐6R and AREG is highly enhanced in airway epithelial cells in response to CS‐induced injury. Next to ADAM17, we highlight the importance of EGFR in the regulation of IL6R and AREG release and mRNA expression. Moreover, CS‐induced ADAM17‐mediated shedding of IL6R and AREG is especially high in COPD ALI‐PBEC, suggesting that reducing ADAM17 activity in COPD might be a potential therapeutic approach.

## Conflict of Interest

Dr. Hiemstra reports receiving research grants from Galapagos NV for the submitted work, and research grants from Boehringer Ingelheim and Grifols outside the submitted work. Dr Scholte reports a research grant from Lexicon inc not related to the submitted work.

## Supporting information




**Figure S1.** The proximity ligation assay (PLA) background signal.

**Figure S2.** Lateral EGFR in ALI‐PBEC, internalized after CS treatment. ALI‐PBEC cultured under basal conditions, including (EGF and PBE) were treated with air or CS as described in the methods section. (A) lateral EGFR immune fluorescence signal (green) becomes more diffuse 3 h after CS treatment. (B) Lateral E‐cadherin (red) illustrates the partial cytoplasmic localisation of EGFR. (C) This is confirmed in a separate experiment, after 10 min exposure to air or CS, with three separate filters each.
