# Supplementary material for: ADAM17 and EGFR regulate IL‐6 receptor and amphiregulin mRNA expression and release in cigarette smoke‐exposed primary bronchial epithelial cells from patients with chronic obstructive pulmonary disease (COPD)
Source: Physiol Rep. 2016 Aug 25;4(16):e12878. doi: 10.14814/phy2.12878 (PMC5002905; doi:10.14814/phy2.12878)
Supplement: Supplementary file 1 — Figure S1. The proximity ligation assay (PLA) background signal. [file PHY2-4-e12878-s001.pdf]

Supplementary Figure S1. The proximity ligation assay (PLA) background signal.

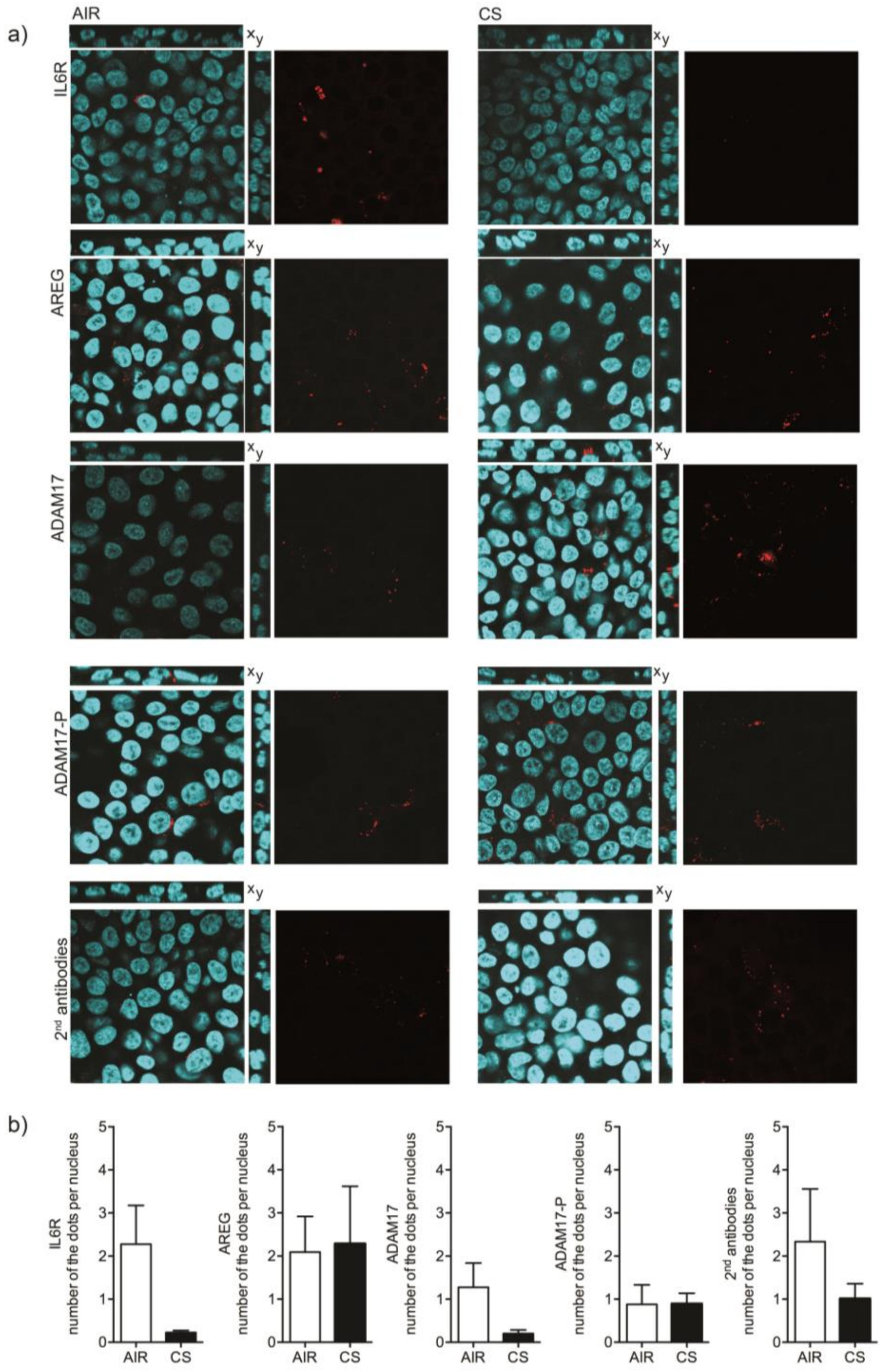

PLA recognizes two proteins that are in close proximity by using a combination of two specific antibodies and two complementary secondary antibodies carrying the ligation probes (Fredriksson et al., 2002). To check the specificity of the PLA signal, the filters with ALI-PBEC used for the assay of Figure 5 (COPD n=1) were incubated with only one of the first antibodies: IL6R, AREG ADAM17, ADAM17PT^{735}, or no first antibodies (2nd antibodies control). (a) The representative immunofluorescence pictures are shown. Left panel shows PLA signal of the apical region (red dots) and right panel presents merged signals of nuclei (blue) and PLA (red) in the x-y sections of the confocal z-stack. (b) The number of dots detected by confocal microscopy in each condition is expressed per nucleus
