# Supplementary material for: ADAM17 and EGFR regulate IL‐6 receptor and amphiregulin mRNA expression and release in cigarette smoke‐exposed primary bronchial epithelial cells from patients with chronic obstructive pulmonary disease (COPD)
Source: Physiol Rep. 2016 Aug 25;4(16):e12878. doi: 10.14814/phy2.12878 (PMC5002905; doi:10.14814/phy2.12878)

Supplementary Figure S2.

Lateral EGFR in ALI-PBEC, internalized after CS treatment. ALI-PBEC cultured under basal conditions, including (EGF and PBE) were treated with air or CS as described in the methods section. **A**: lateral EGFR immune fluorescence signal (green) becomes more diffuse three hours after CS treatment **B**: Lateral E-cadherin (red) illustrates the partial cytoplasmic localisation of EGFR. **C:** This is confirmed in a separate experiment, after 10 minutes exposure to air or CS, with three separate filters each.

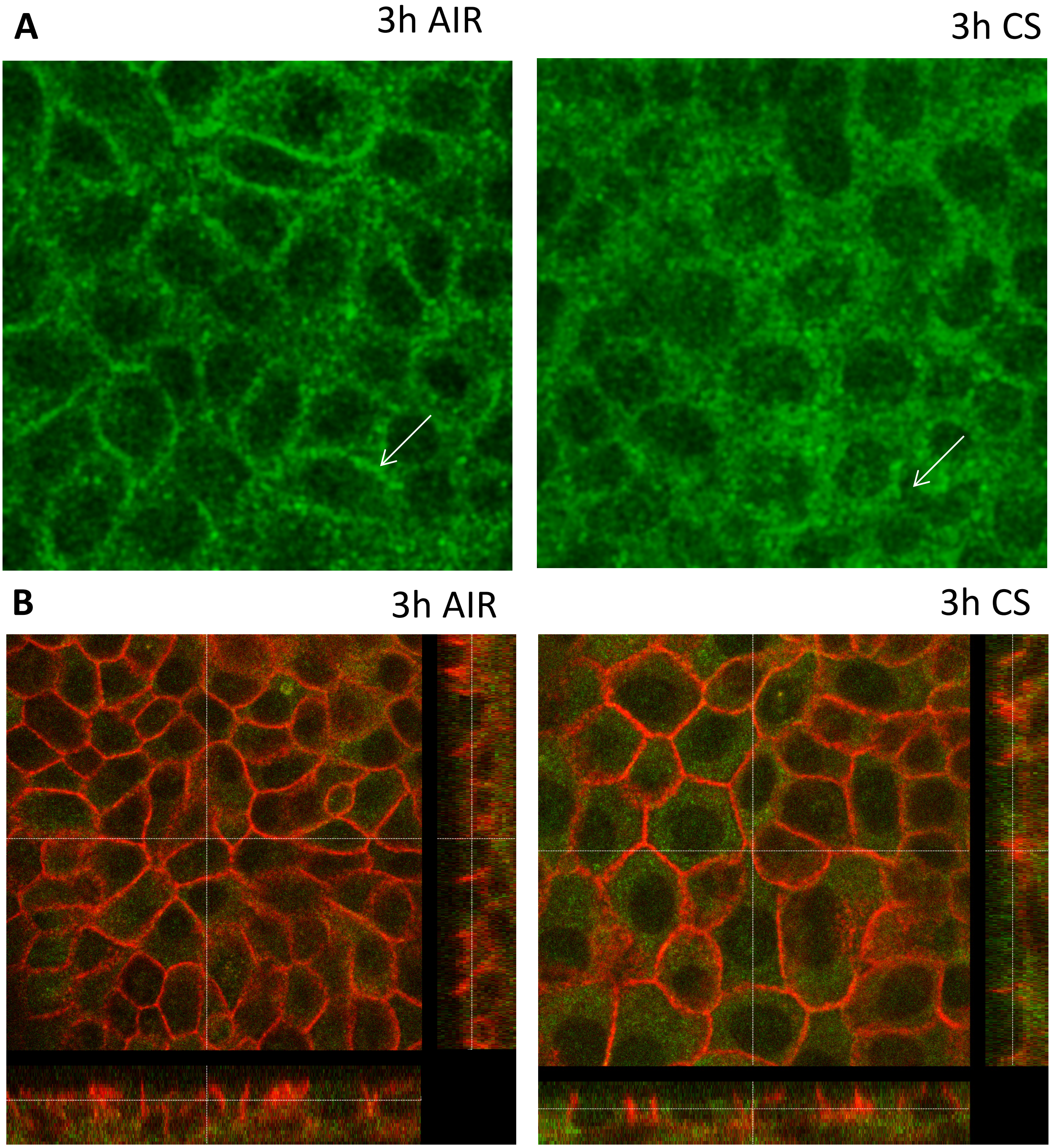

EGFR- green, E-CAD – red; 63x, 1.6 zoom

C

HBEC-ALI 10 minutes Air | HBEC-ALI 10 minutes

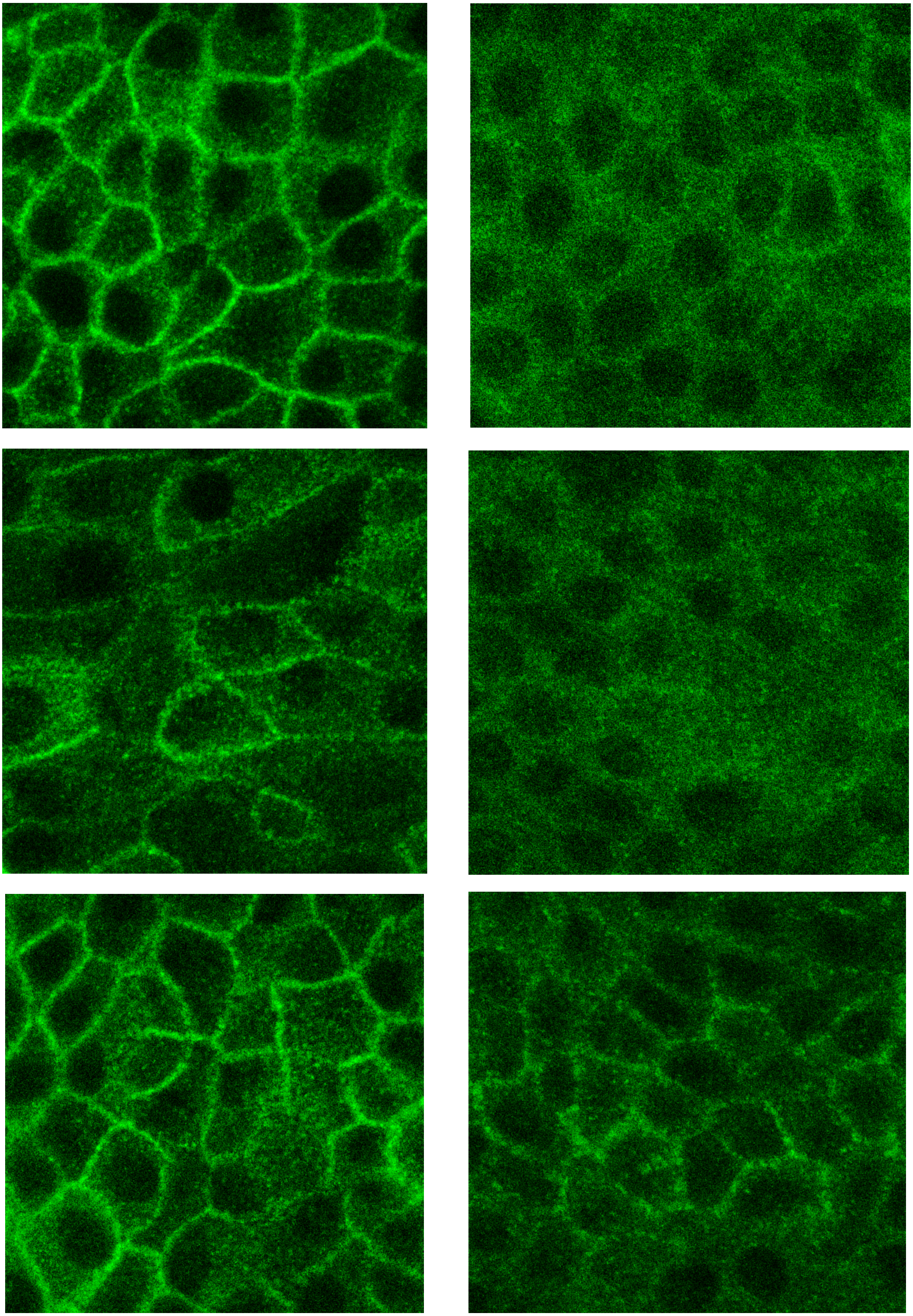

Supplement: Supplementary file 2 — Figure S2. Lateral EGFR in ALI‐PBEC, internalized after CS treatment. ALI‐PBEC cultured under basal conditions, including (EGF and PBE) were treated with air or CS as described in the methods section. (A) lateral EGFR immune fluorescence signal (green) becomes more diffuse 3 h after CS treatment. (B) Lateral E‐cadherin (red) illustrates the partial cytoplasmic localisation of EGFR. (C) This is confirmed in a separate experiment, after 10 min exposure to air or CS, with three separate filters each. [file PHY2-4-e12878-s002.pdf]
